# p85α promotes nucleolin transcription and subsequently enhances EGFR mRNA stability and EGF-induced malignant cellular transformation

**DOI:** 10.18632/oncotarget.7674

**Published:** 2016-02-24

**Authors:** Qipeng Xie, Xirui Guo, Jiayan Gu, Liping Zhang, Honglei Jin, Haishan Huang, Jingxia Li, Chuanshu Huang

**Affiliations:** ^1^ Zhejiang Provincial Key Laboratory for Technology and Application of Model Organisms, School of Life Sciences, Wenzhou Medical University, Wenzhou, Zhejiang, 325035, China; ^2^ Nelson Institute of Environmental Medicine, New York University School of Medicine, Tuxedo, NY 10987, USA

**Keywords:** PI3K, p85α, EGFR, nucleolin, cell transformation

## Abstract

p85α is a regulatory subunit of phosphatidylinositol 3-kinase (PI3K) that is a key lipid enzyme for generating phosphatidylinositol 3, 4, 5-trisphosphate, and subsequently activates signaling that ultimately regulates cell cycle progression, cell growth, cytoskeletal changes, and cell migration. In addition to form a complex with the p110 catalytic subunit, p85α also exists as a monomeric form due to that there is a greater abundance of p85α than p110 in many cell types. Our previous studies have demonstrated that monomeric p85α exerts a pro-apoptotic role in UV response through induction of TNF-α gene expression in PI3K-independent manner. In current studies, we identified a novel biological function of p85α as a positive regulator of epidermal growth factor receptor (EGFR) expression and cell malignant transformation *via* nucleolin-dependent mechanism. Our results showed that p85α was crucial for EGFR and nucleolin expression and subsequently resulted in an increase of malignant cellular transformation by using both specific knockdown and deletion of p85α in its normal expressed cells. Mechanistic studies revealed that p85α upregulated EGFR protein expression mainly through stabilizing its mRNA, whereas nucleolin (NCL) was able to bind to egfr mRNA and increase its mRNA stability. Consistently, overexpression of NCL in p85α−/− cells restored EGFR mRNA stabilization, protein expression and cell malignant transformation. Moreover, we discovered that p85α upregulated NCL gene transcription *via* enhancing C-Jun activation. Collectively, our studies demonstrate a novel function of p85α as a positive regulator of EGFR mRNA stability and cell malignant transformation, providing a significant insight into the understanding of biomedical nature of p85α protein in mammalian cells and further supporting that p85α might be a potential target for cancer prevention and therapy.

## INTRODUCTION

The epidermal growth factor receptor (EGFR), also called ErbB1, is first identified member of the subfamily of tyrosine kinase receptors [1]. The ligands of EGFR include EGF, TGFα, amphiregulin, heparin-binding EGF-like factor (HB-EGF), betacellulin (BTC) and epiregulin [2–4]. Ligand binding and activation leads to EGFR dimerization and auto-phosphorylation of tyrosine residues in the C-terminal region that provide docking sites for Src homology 2 or phosphotyrosine-binding domain-containing signaling molecules [5]. The EGFR downstream regulated signaling pathways include PI3K/Akt axis and Ras/Raf/MAPK (ERK, JNK and p38) axis [6]. EGFR plays an important role in extensive crosstalk among multiple signaling pathways and regulation of various cell functions [7]. EGFR also plays a significant role in tumor development and progression, including cell proliferation, regulation of apoptotic cell death, angiogenesis and metastatic spread. In most cell types, EGFR is found in amounts varying from 2 × 10^4^ to 2 × 10^5^ receptors per cell. Overexpression of EGFR up to > 10^6^ receptors per cell has been described for many cancer types, such as in the lung, head and neck, colon, pancreas, breast, ovary, bladder and kidney, and in gliomas [8–11]. Moreover, several studies demonstrate that EGFR expression correlates with the reduced disease-free and overall survival, poor prognosis, increased risk of disease recurrence, advanced tumor stage, and increased risk of metastasis [12].

EGFR expression in human tissues could be regulated at levels of gene amplification, mRNA transcription and degradation, protein translation and degradation [13, 14]. EGFR mRNA is central to the flow of genetic information, and regulation of mRNA stability is a powerful mechanism for altering gene expression, and is regulated by multiple proteins [15–17]. Microarray analyses suggest that approximately 40%–50% of changes in gene expression in response to extracellular treatment occurred due to altered mRNA stability [18, 19]. The defect in regulation of mRNA stability might lead to complicated disorders, including cancers [18].

The class I phosphoinositide 3-kinase (PI3K) is heterodimer lipid enzyme that is composed of a catalytic (p110) and a regulatory (p85) subunit. Upon activation, PI3K produces a key second messenger lipid, phosphatidylinositol 3, 4, 5-trisphosphate (PIP3), and regulates many cellular functions, such as cell growth and survival [20, 21]. The regulatory p85 subunit has five variants (p85α, p55α, p50α, p85β, and p55γ) [20, 22]. Of these isoforms, p85α is predominantly and ubiquitously expressed in most tissues and is thought to be the major element of response to most stimuli [23]. In addition to forming a complex with the p110 catalytic subunit, p85α also exists in a monomeric form due to the greater abundance of p85α than of p110 in many cell types [24]. The monomeric p85α is able to act as a mediator for transducing the insulin-like growth factor 1-dependent cellular response [25] and is also involved in the apoptotic response under oxidative stress in a PI3K-independent manner. Our previous studies demonstrate that p85α mediates apoptotic response following UV radiation in a PI3K-independent manner [26]. However, the role of p85α alone in regulation of EGFR expression and its related mechanisms has not been explored yet. Here we reported that p85α was able to regulate EGFR expression by increasing in egfr mRNA stability and EGF-induced cell malignant transformation. We further showed that NCL expression mediated by p85α was able to bind with egfr mRNA, which protected egfr mRNA from degradation.

## RESULTS

### p85α was essential for EGFR expression and EGF-induced cell transformation

**Figure 1:**
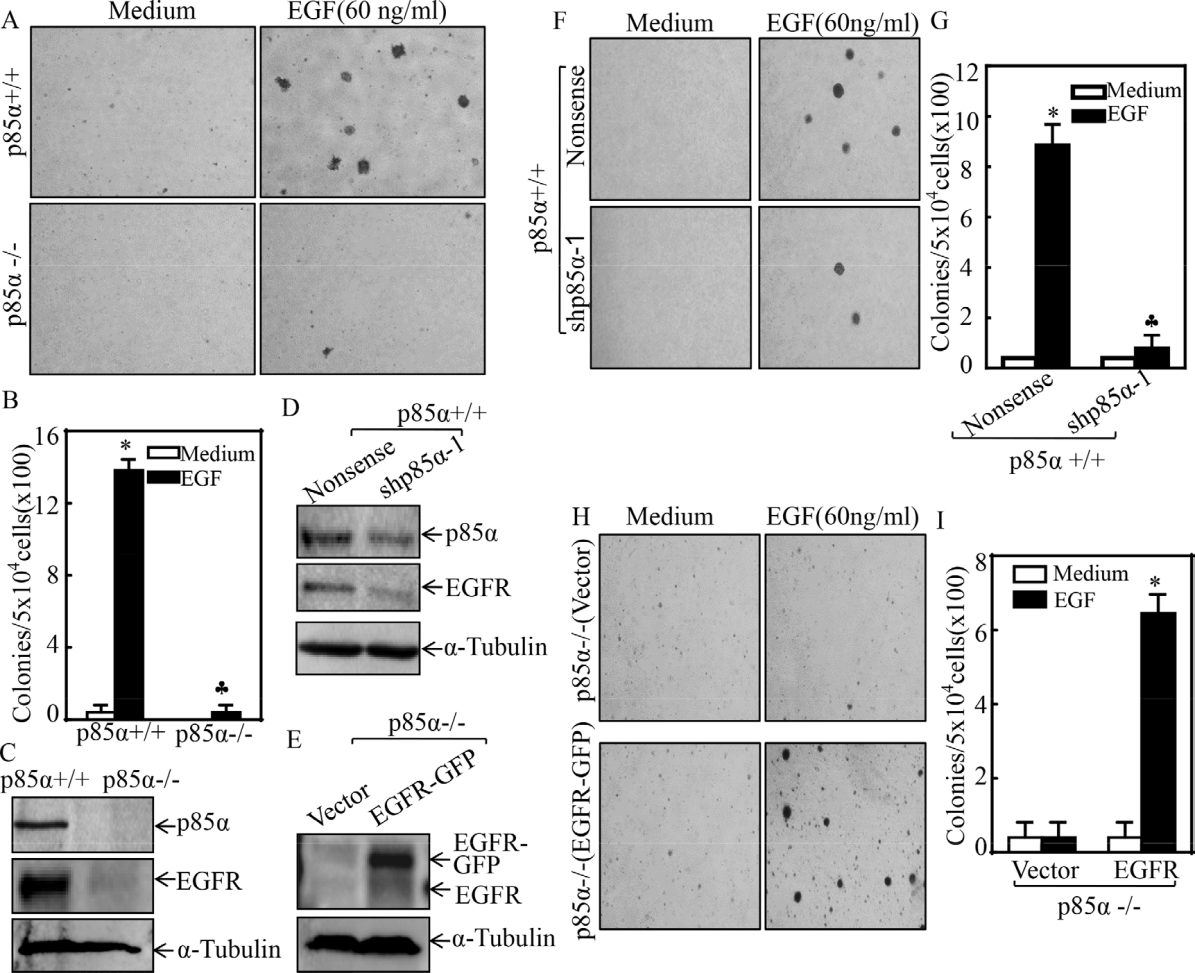
p85α was required for EGFR expression and EGF-induced malignant cell transformation (**A & B**) 5 × 10^4^ cells of p85α+/+ and p85α−/− cells were subjected to soft agar assay in presence of EGF (60 ng/ml). The images were captured under inverted microscopies after being incubated in a 37°C with 5% CO_2_ incubator for 3 weeks (A) and the colonies were also counted (B). Each bar indicates the mean ± SD from triplicate assays. The symbol (*) indicates a significant increase as compared with the medium control, while the symbol (♣) indicates a significant decrease in comparison to p85α+/+ cells (*P* < 0.05). (**C**–**E**) the cells as indicated were seeded into 6-well plates. The cells were extracted upon the density reaching 80–90%, and the cell extracts were subjected to Western Blot with indicated antibodies. α-Tubulin was used as protein loading controls. (**F–I**) the indicated cell transfectants were subjected to soft agar assay in presence of 60 ng/ml EGF same as described in “A & B”. Each bar indicates the mean ± SD from triplicate assays. The symbol (*) indicates a significant increase as compared with the medium control, while the symbol (♣) indicates a significant decrease in comparison to p85α+/+ (Nonsense) (G) or p85α−/− (Vector) (I) (*P* < 0.05).

Many tumors exhibit an increased activation of PI3K signaling pathway, while p85α regulates multiple cellular biological functions either through PI3K-dependent or -independent manners [25, 26]. To determine the role of p85α in the regulation of cell transformation, we first utilized EGF as a tumor promoter to establish an EGF-induced cell malignant transformation experimental system [27] and consequently evaluated EGF-induced anchorage-independent growth abilities in p85α+/+ and p85α−/− cells. The results showed that knockout of p85α led to a completely deficiency of anchorage-independent growth upon EGF exposure in comparison to that in p85α+/+ cells under same experimental conditions (Figure 1A and 1B), suggesting that p85α was crucial for EGF-induced cell malignant transformation. To elucidate the molecular mechanism underlying p85α regulation of EGF-induced cell transformation, we determined EGFR protein expression in both p85α+/+ and p85α−/− cells. EGFR protein expression was found to be almost completely impaired in p85α−/− cells (Figure 1C), which is consistent with the cell responses of anchorage-independent growth abilities following EGF treatment and further revealing that p85α is critical for EGFR expression in addition to its requirement for cell malignant transformation after EGF treatment. This notion was greatly strengthened by the results obtained from using a specific short hairpin RNAs (shRNAs) targeting p85α to knockdown its expression in p85α+/+ cells. As shown in Figure 1D, knockdown of p85α expression by its shRNA impaired EGFR expression as compared with their scramble control transfectants, p85α+/+ (Nonsense). Moreover, we constructed a pEGFP-EGFR and stably transfected it into p85α−/− cells to restore EGFR expression. The stable transfectants p85α−/− (EGFR-GFP) and its scramble control p85α−/− (Vector) were established as shown in Figure 1E. The results from determination of EGF-induced transformation indicated that knockdown of p85α in p85α+/+ (shp85α-1) cells significantly attenuated the cell malignant transformation upon EGF (60 ng/ml) treatment compared with p85α+/+ (Nonsense) cells (Figure 1F and 1G), whereas overexpression of EGFR-GFP in p85α−/− (EGFR-GFP) cells profoundly promoted the cell malignant transformation upon EGF treatment in comparison to that in p85α−/− (Vector) (Figure 1H and 1I). These results demonstrate that EGFR serves as an important downstream mediator responsible for p85α promoting cell transformation following EGF treatment.

### p85α mediated EGFR mRNA stabilization

**Figure 2:**
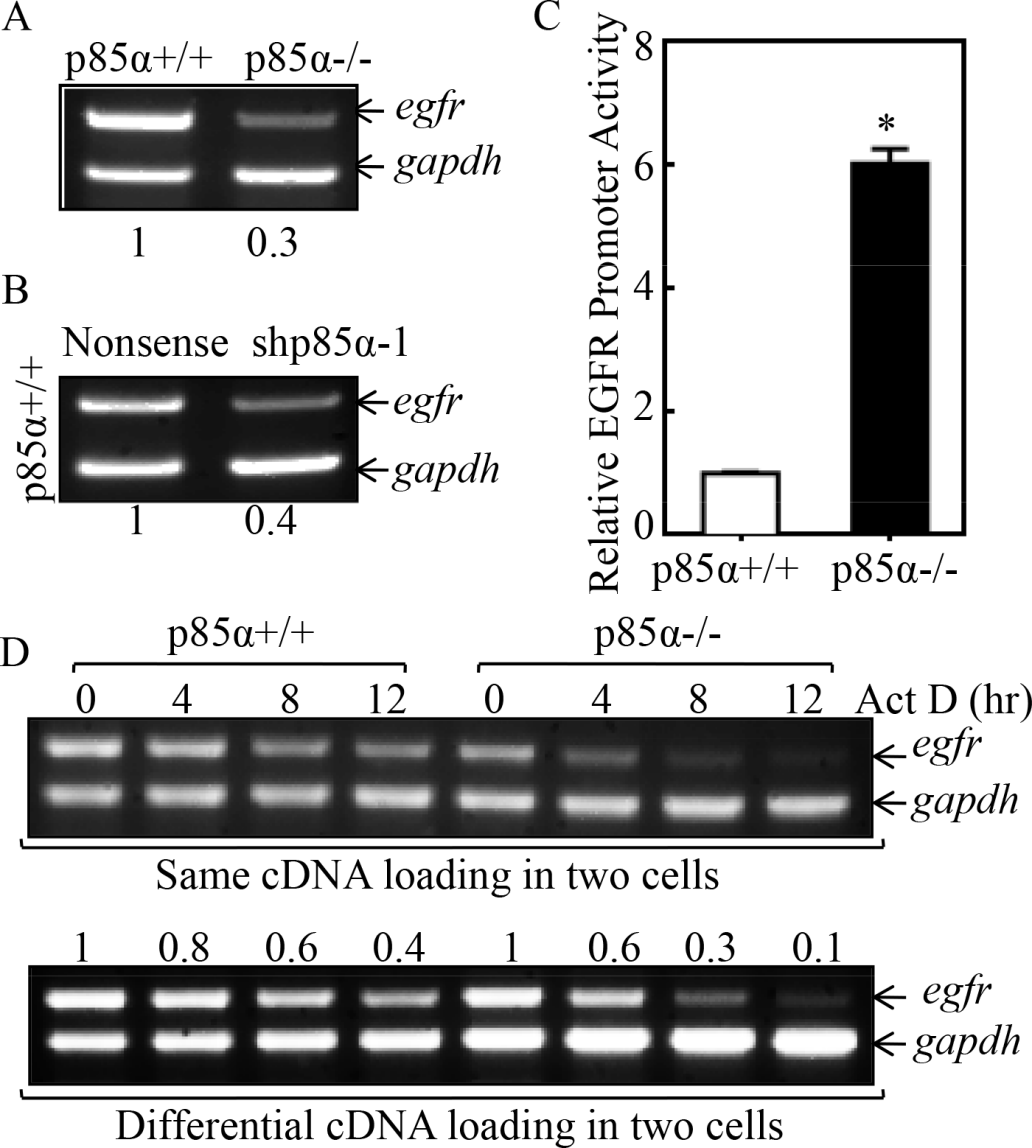
p85α mediated EGFR mRNA stabilization (**A & B**) p85α+/+, p85α−/− cells and their transfectants, were seeded into 6-well plates. The cells were extracted with Trizol reagent for the total RNA isolation upon the density reaching 80–90%. *Egfr* mRNA were determined with RT-PCR by using the specific primers. *Gapdh* was used as an internal control. (**C**) p85α+/+ and p85α−/− cells transfected with EGFR promoter-driven luciferase reporter together with pRL-TK were seeded into 96-well plates. After being cultured twenty-four hours, the luciferase activity was measured and pRL-TK was used as an internal control to normalize the transfection efficiency. The results were presented as luciferase activity relative to p85α+/+ cells (Relative EGFR Promoter Activity). Each bar indicates the mean ± SD of three replicate wells. The symbol (*) indicates a significant increase as compared with p85α+/+ (Nonsense) (*P* < 0.05). (**D**) p85α+/+ and p85α−/− cells were seeded into 6-well plates. After synchronization, p85α+/+ and p85α−/− cells were treated with Actinomycin D (Act D) for the indicated time points, then total RNA was isolated and subjected to RT-PCR analysis for mRNA levels of *Egfr* and *Gapdh*.

EGFR expression is delicately regulated at multiple levels, including transcriptional, post-transcriptional, translational, and post-translational levels [28]. To define the mechanism underlying p85α regulation of EGFR expression, we first compared EGFR mRNA levels between p85α+/+ and p85α−/− cells, and we found that EGFR mRNA expression was profoundly downregulated in p85α−/− cells as compared with that in p85α+/+ cells (Figure 2A). This finding was further supported by the results obtained from utilization of shRNA specific targeting p85α, showing that EGFR mRNA expression was dramatically inhibited in p85α knockdown transfectant, p85α+/+ (shp85α-1), in comparison to the scramble transfectant, p85α+/+ (Nonsense) (Figure 2B). To test whether p85α regulated EGFR mRNA transcription, EGFR promoter-driven luciferase reporter was transfected into both p85α+/+ cells and p85α−/− cells and the promoter transcription activity was compared between the two transfectants. As shown in Figure 2C, opposite to EGFR mRNA expression, EGFR promoter-driven luciferase reporter transcription activity in p85α−/− cells was significant higher than that observed in p85α+/+ cells (Figure 2C), excluding the possibility that p85α positively regulates EGFR mRNA transcription. Thus, we next determined the possibility of p85α regulation of EGFR mRNA stability. The p85α+/+ and p85α−/− cells were treated with the de novo mRNA synthesis inhibitor actinomycin D (Act D), and the decay rate of EGFR mRNA was assessed by RT-PCR (Top panel of Figure 2D). To made the comparable of mRNA levels between p85α+/+ and p85α−/− cells, we loaded more total cDNA in all samples of p85α−/− cells for RT-PCR than those in p85α+/+ cells (as seen in gadph levels of bottom panel). As shown in Figure 2D, EGFR mRNA stability was dramatically reduced in p85α−/− cells as compared with that observed in p85α+/+ cells. Our results indicate that p85α is crucial for EGFR mRNA stabilization.

### Nucleolin (NCL), but not HUR, was responsible for p85α-mediated EGFR mRNA stabilization

**Figure 3:**
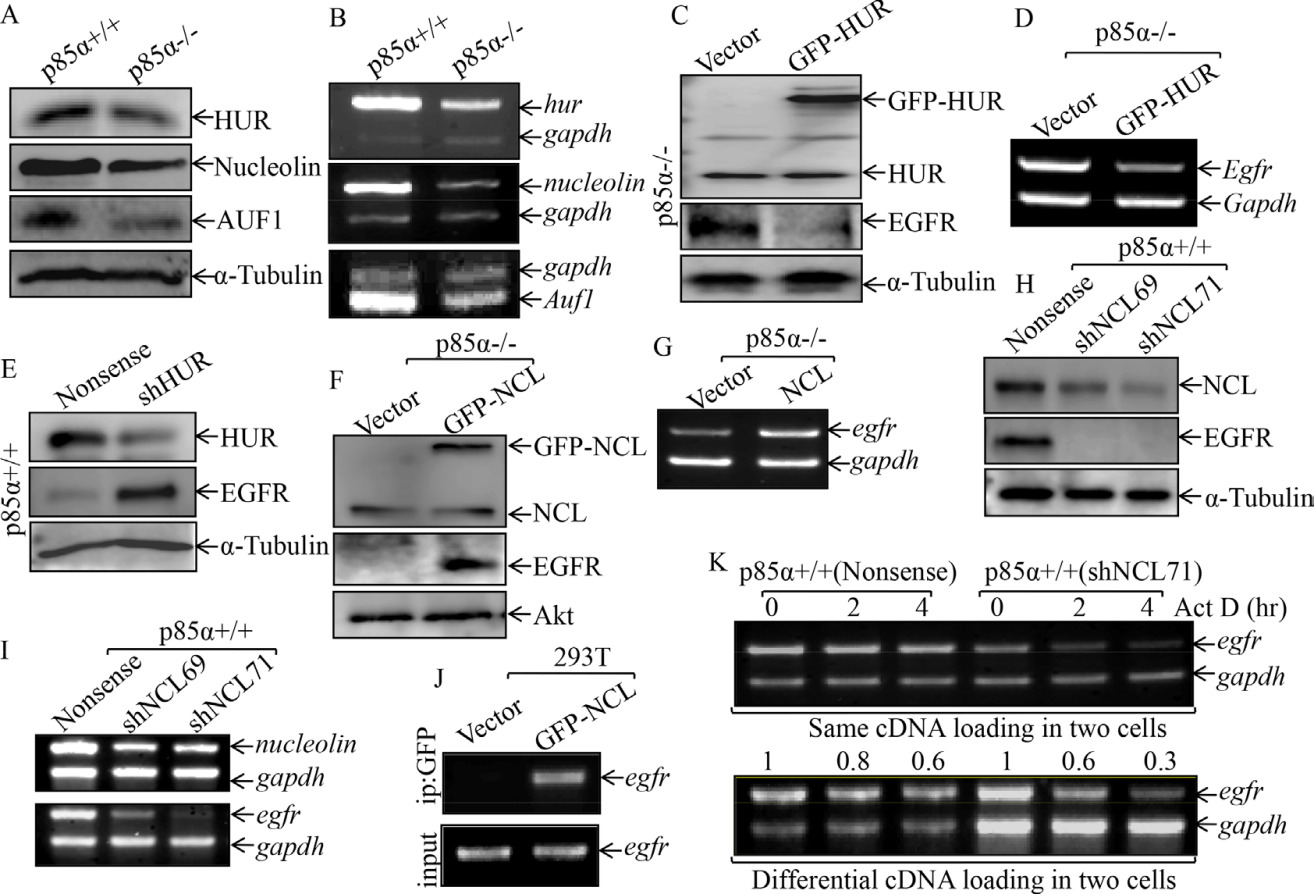
NCL, but not HUR, mediated p85α stabilization of EGFR mRNA (**A & B**) p85α+/+ and p85α−/− cells, as well as their transfectants, were cultured in 6-well plates till cell density reaching 80–90%, and then extracted for either whole cell extracts or total RNA. Western blot was carried out for determination of the indicated protein expression with specific antibodies and α-Tubulin was used as a control for protein loading (**A, C, E, F & H**); RT-PCR was used to determine indicated mRNA expression and gadph was used as an internal control (**B, D, G, & I**). (**J**) 293T cells were transiently transfected with either GFP-NCL or its control vector. After the cell density reaching 80-90%, the cells were extracted and RNA-IP assay was carried out with specific primer of *egfr*. (**K**) the p85α+/+ (Nonsense) or p85α+/+ (shNCL71) were cultured till the cell density reaching 80–90%, and then treated with Actinomycin D (Act D) for the indicated time points. The total RNA was isolated and subjected to RT-PCR analysis for determination of mRNA levels of *Egfr* and *Gapdh*.

The degradation of mRNAs can be modulated *via* cis-acting sequence elements or trans-acting factors [29, 30]. Several RNA-binding proteins, such as nucleolin (NCL), HUR, and AUF1, have been reported to bind their target mRNA and modulate mRNA stability [31–33]. Thus, we tested whether those RNA-binding proteins were involved in the p85α upregulation of EGFR mRNA stability. As exhibited in Figure 3A, the downregulation of HUR, NCL and AUF1 protein expression were observed in p85α−/− cells as compared with those in p85α+/+ cells. Consistently, the mRNA levels of HUR, NCL, and AUF1 were also reduced in p85α−/− cells (Figure 3B). Given that AUF1 can function as mRNA destabilizers when bound to an ARE-containing mRNA [34], AUF1 was excluded as a p85α downstream effector being mediating p85α stabilization of EGFR mRNA. Since HUR has been reported to stabilize its binding mRNA [35], we tested potential role of HUR in p85α regulation of EGFR mRNA stability by introduction of pEGFP-HUR into in p85α−/− cells. As shown in Figure 3C, the stable transfectants p85α−/− (GFP-HUR) and its scramble control p85α−/− (Vector) cells were established and identified. Ectopic expression of GFP-HUR cells dramatically inhibited EGFR mRNA and protein expression in p85α−/− (Figure 3C and 3D). Moreover, the results obtained from using specific short hairpin RNAs (shRNAs) targeting HUR to knockdown its expression in p85α+/+ cells, consistently showed that HUR is a negative regulator, rather than positive regulator, for EGFR mRNA stability (Figure 3E). We, therefore, next investigated the potential role of NCL in regulation of EGFR mRNA stability. The pEGFP-NCL plasmid was stably transfected into p85α−/− cells and stable transfectants p85α−/− (GFP-NCL) and its scramble control p85α−/− (Vector) were identified (Figure 3F). EGFR protein and mRNA expression was markedly increased in p85α−/− (GFP-NCL) cells as compared with those observed in p85α−/− (Vector) (Figure 3F and 3G). Moreover, knockdown of nucleolin by its specific shRNAs in p85α+/+ cells dramatically reduced EGFR protein and mRNA expression (Figure 3H and 3I). These results reveal that NCL can stabilize EGFR mRNA. To test whether nucleolin is able to bind to EGFR mRNA, RNA-IP assay was carried out in which anti-GFP antibody was used to pull down all mRNAs that physically interacted with GFP-NCL protein. The mRNA was then extracted from the precipitated complex and reverse transcription-PCR was performed to detect the presence of EGFR mRNA. As shown in Figure 3J, EGFR mRNA was found to be specific present in the immune-complex of cell extracts of 293T(GFP-NCL), but not in 293T (Vector), strongly indicating that nucleolin indeed interacts with EGFR mRNA. We further compared the egfr mRNA degradation rates between p85α+/+ (shNCL71) and p85α+/+ (Nonsense) cells (Figure 3K). To validate the role of nucleolin in stabilizing EGFR mRNA, p85α+/+ (Nonsense) and p85α+/+ (shNCL71) cells were treated with the de novo mRNA synthesis inhibitor actinomycin D (Act D), and the decay rate of EGFR mRNA was assessed by RT-PCR. To made the comparable of mRNA levels between p85α+/+ and p85α−/− cells, we loaded more total cDNA in all samples of p85α+/+ (shNCL71) cells for RT-PCR than those in p85α+/+ (Nonsense) cells (as seen in gadph in bottom panel of Figure 3K). As shown in Figure 3K, EGFR mRNA stability was dramatically reduced in p85α+/+ (shNCL71) transfectants in comparison to that in p85α+/+ (Nonsense) cells. Our results clearly demonstrate that nucleolin is p85α downstream mediator being responsible for binding to EGFR mRNA for positive regulation of its stability.

### NCL was critical for p85α promotion of EGF-induced cell transformation

**Figure 4:**
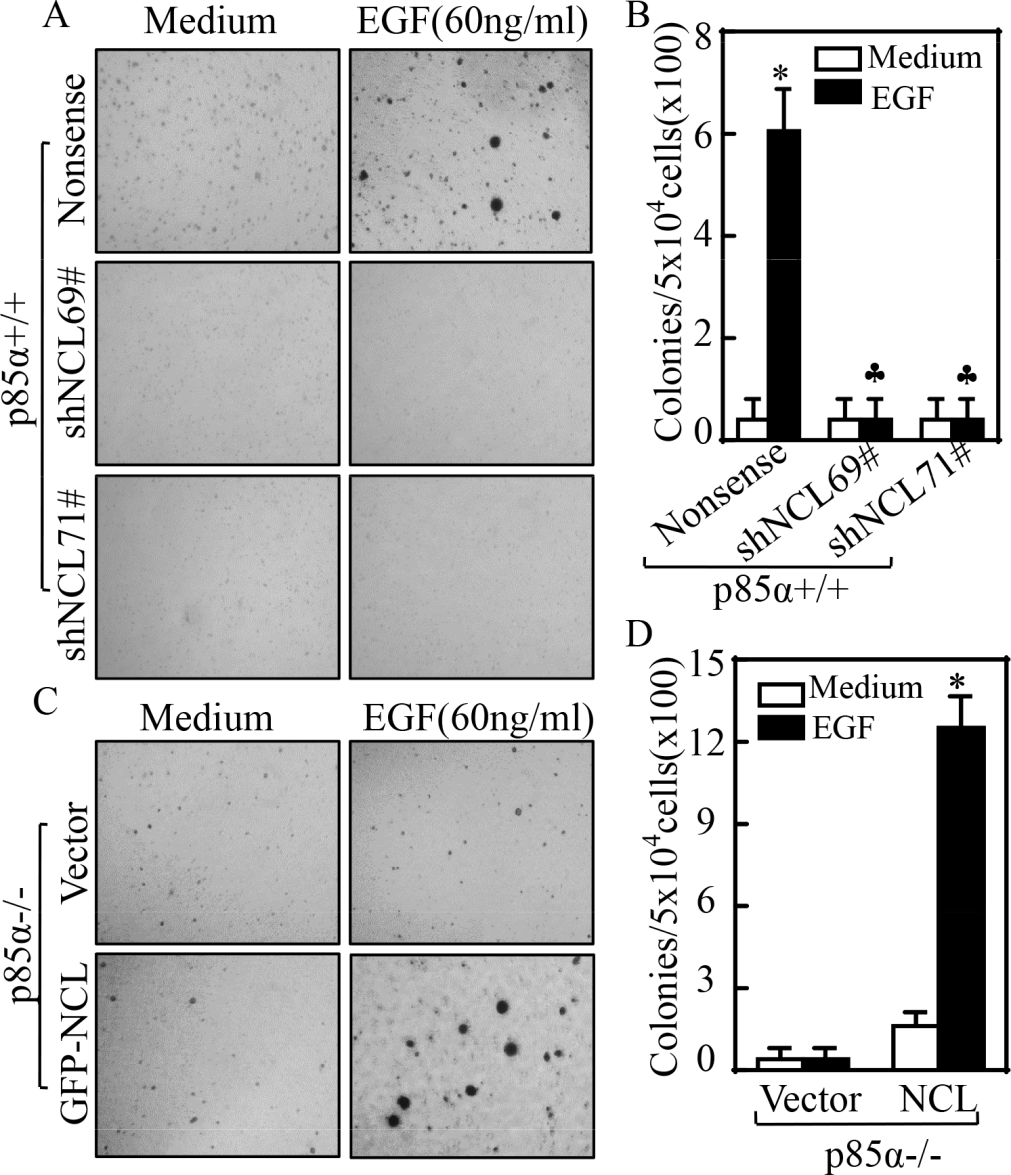
NCL is critical for p85α-regulated EGF-induced malignant cell transformation 5 × 10^4^ of stable transfectants of p85α+/+ and p85α−/− as indicated were subjected to soft agar assay in presence of 60 ng/ml EGF. The images were captured under inverted microscopies after being incubated in a 37°C with 5% CO_2_ incubator for 3 weeks (**A & C**) and the colonies were counted (**B & D**). Each bar indicates the mean ± SD from triplicate assays. (B) The symbol (*) indicates a significant increase as compared with the medium control, while the symbol (♣) indicates a significant decrease in comparison to p85α+/+ (Nonsense) cells (*P* < 0.05). The symbol (*) indicates a significant increase as compared with p85α−/− (Vector) (*P* < 0.05) (D).

Our above results showed that NCL could bind to and stabilize EGFR mRNA. Thus, we knocked down NCL in p85α+/+ cells to investigate its effects on cell transformation following EGF treatment. As shown in Figure 4A and 4B, knockdown of NCL in p85α+/+ cells, p85α (shNCL69) and p85α+/+ (shNCL71), dramatically inhibited the malignant cell transformation upon EGF treatment as compared with that in p85α+/+ (Nonsense) under same experimental conditions. Moreover, we overexpressed NCL in p85α−/− cells and the results showed that NCL ectopic overexpression, p85α−/− (GFP-NCL) cells, restored the malignant cell transformation capability in comparison to that in p85α−/− (Vector) cells following EGF treatment (Figure 4C and 4D). These results demonstrate that nucleolin is critical for p85α mediation of cell transformation following EGF treatment.

### p85α upregulated NCL transcription through c-Jun-dependent axis

**Figure 5:**
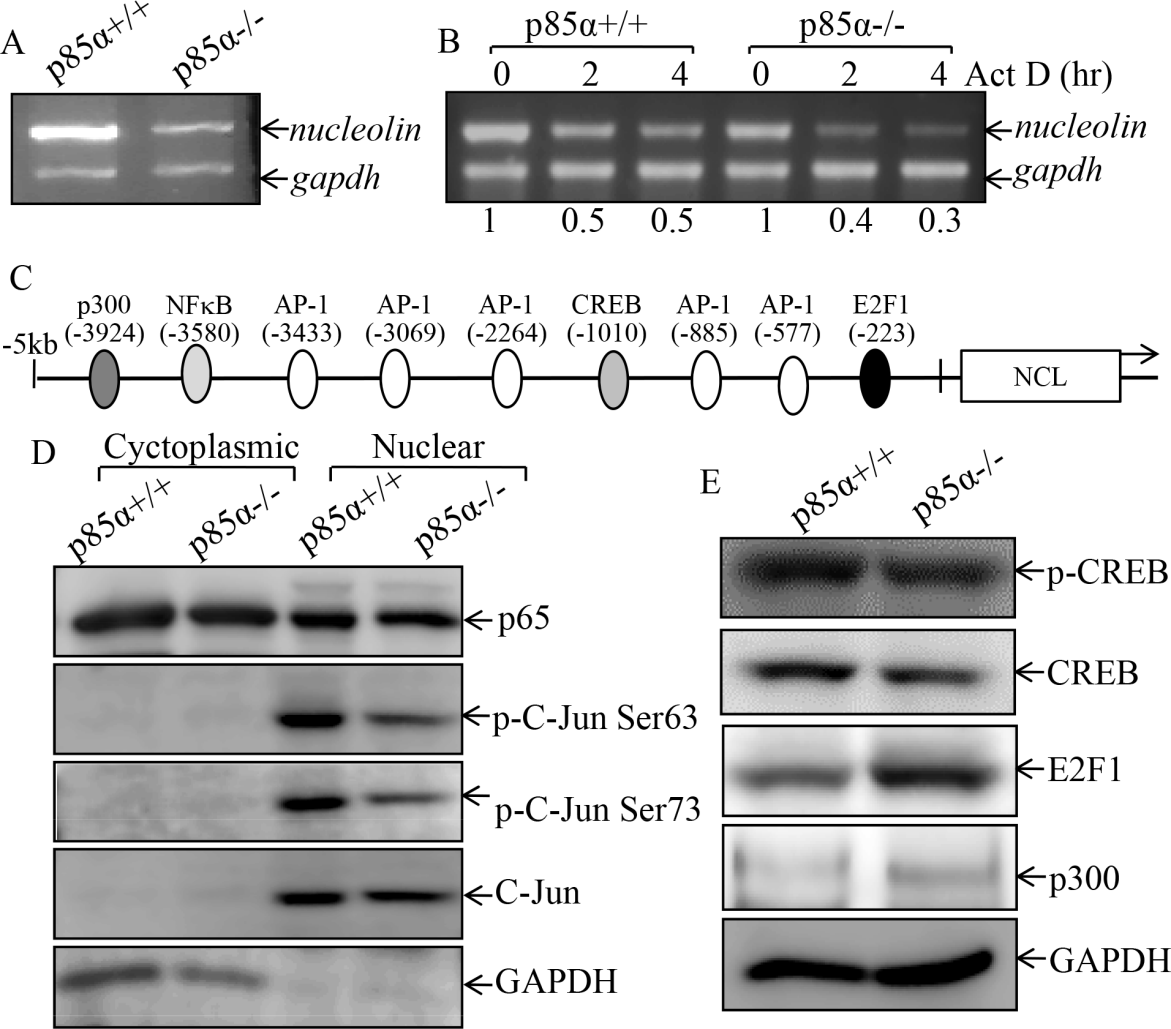
p85α regulated NCL transcription and C-Jun activation (**A**) p85α+/+ and p85α−/− cells were cultured in 6-well plates till cell density reaching 80–90%, and then extracted for total RNA with Trizol reagent. RT-PCR was used to determine nucleolin mRNA expression, while gadph was used as an internal control, (**B**) p85α+/+ and p85α−/− cells were cultured in 6-well plates till cell density reaching 80–90%, and the cells were then treated with Act D for the indicated time points, and were then used for total RNA isolation. The total RNA was subjected to RT-PCR for determination of mRNA levels of Egfr and Gapdh. (**C**) Potential transcriptional factor binding sites in NCL promoter region (−5000-+1) analyzed by using the TRANSFAC 8.3 engine online. (**D**) p85α+/+ and p85α−/− cells were cultured in 6-well plates till cell density reaching 80–90%, the cells were extracted and the whole cell extracts were used to isolate cytoplasmic and nuclear fractions according to the protocol of the nuclear/cytosol fractionation kit. The isolated protein fractions were subjected to Western blot. GAPDH were used as control for protein loading. (**E**) p85α+/+ and p85α−/− cells were cultured in 6-well plates till cell density reaching 80–90%, the cells were extracted and cell extracts were subjected to Western blot for determination of the indicated protein expression with specific antibodies and GAPDH was used as protein loading controls.

**Figure 6:**
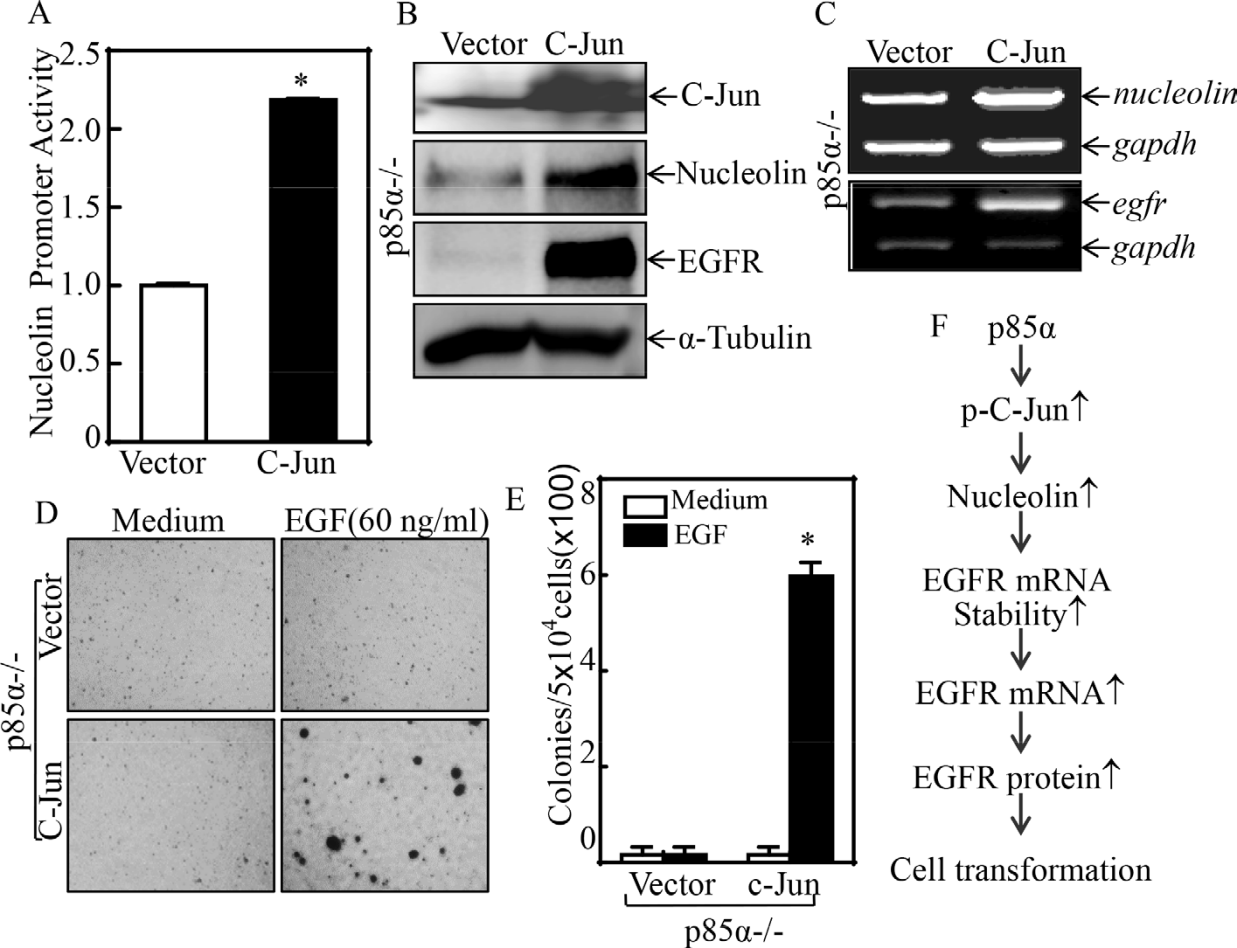
C-Jun mediated nucleolin transcription and EGF-induced cell transformation (**A**) p85α−/− (Vector) and p85α−/− (C-Jun) were transfected with nucleolin promoter-driven luciferase reporter together with pRL-TK. The transfectants were seeded into 96-well plates for determination of nucleolin promoter activity by measuring luciferase activity. pRL-TK was used as an internal control to normalize the transfection efficiency. Each bar indicates the mean ± SD from three replicate assays. (**B & C**) p85α−/− (Vector) and p85α−/− (C-Jun) were extracted for either whole cell protein extracts or total RNA. Whole cell extracts were subjected to Western blot for determination of the indicated protein expression with specific antibodies and α-Tubulin was used as protein loading controls (B). Total RNA was subjected to RT-PCR for determination the indicated mRNA expression, and gadph was used as an internal control (C). (**D & E**) 5 × 10^4^ of stable transfectants, p85α−/− (Vector) and p85α−/− (C-Jun), were subjected to soft agar assay in presence of 60 ng/ml EGF. The images were captured under inverted microscopies after being incubated in a 37°C with 5% CO_2_ incubator for 3 weeks (D) and the colonies were also counted (E). Each bar indicates the mean ± SD from triplicate assays. The symbol (*) indicates a significant increase as compared with p85α−/− (Vector) (*P* < 0.05). (**F**), Novel molecular mechanism underlying p85α regulation of EGFR expression and malignant cell transformation followed EGF treatment.

Given our results showing that NCL is important for p85α regulation of EGFR expression and EGF-induced malignant cell transformation, our subsequent efforts were given to the mechanisms being responsible for p85α upregulation of NCL. NCL expression has been reported to be regulated at multiple levels, including transcription, post-transcription, translation, and post-translation [36]. We, therefore, examined the NCL mRNA expression in p85α+/+ and p85α−/− cells. The results indicated the NCL mRNA were markedly decreased in p85α−/− cells in comparison to that in p85α+/+ cells (Figure 5A). The results obtained from evaluation of NCL mRNA stability revealed that NCL mRNA degradation rates are only show slightly difference between p85α+/+ and p85α−/− cells (Figure 5B), suggesting that p85α might regulate NCL transcription. To test this notion, TFANSFAC^®^ Transcription Factor Binding Sites Software (Biological Database, Wolfenbüttel, Germany) was applied for bioinformatics analysis of the NCL promoter. The results indicated that the mouse NCL gene promoter region contains the putative DNA-binding site of p300, nuclear factor κB (NF-κB), C-Jun, CREB-binding protein (CBP), and E2F1 (Figure 5C). We next determined the role of p85α in regulation of those transcription factor expression and/or nuclear translocation in both p85α+/+ and p85α−/− cells. As shown in Figure 5D and 5E, inhibition of p-C-Jun Ser63 and p-C-Jun Ser73 protein expression was observed in the nuclear protein extract of p85α−/− cells, while there was no markedly inhibition of NF-κB (p65), C-Jun and CREB. Given that E2F1 and p300 both can promote the transcription of target gene [37, 38] and they increased in p85α−/− cells, E2F1 and p300 were excluded as a p85α downstream effector being mediating the transcription of nucleolin. To define the role of C-Jun in p85α-mediated nucleolin transcription, we co-transfected nucleolin promoter-driven luciferase reporter with C-Jun into p85α−/− cells and the effect of C-Jun overexpression on nucleolin transcription was evaluated. The results showed that ectopic expression of C-Jun resulted in increasing of nucleolin promoter transcriptional activity (Figure 6A). Consistently, protein and mRNA expression of Nucleolin and EGFR was also profoundly upregulated in p85α−/− cells (Figure 6B & 6C). Moreover, overexpression of C-Jun also significantly rescued malignant cell transformation ability of p85α−/− cells following EGF treatment (Figure 6D & 6E). Our results conclusively demonstrate that C-Jun mediates nucleolin transcription and expression, which subsequently upregulating EGFR mRNA stability, and in turn promoting EGF-induced malignant cell transformation as summarized in Figure 6F.

## DISCUSSION

Although the deregulation of phosphatidylinositol 3-kinase (PI3K) and its regulatory unit p85α has been reported in many human cancers [39, 40], the mechanisms for their action in cancer development are far away from understood. EGFR is overexpressed in many aggressive cancers, and previous study indicates that p85α can be activated by transmembrane tyrosine kinase receptors, such as EGFR, HER2 and IGF1-R [41]. However, the role of p85α in regulation of EGFR expression has never been elucidated. Here we found that p85α has a positive regulatory effect on EGFR expression, and this positive effect of p85α is regulated by NCL-dependent increased EGFR mRNA stability and EGFR protein expression. Further studies found that p85α is crucial for C-Jun-initiated NCL transcription, which can bind to EGFR mRNA and inhibit its mRNA degradation, by which p85α promote malignant cell transformation following EGF treatment. Our findings that p85α regulates EGFR mRNA stability and cell transformation *via* initiating C-Jun/nucleolin axis provide a new insight into the understanding of natural face of p85α in regulation of multiple cellular function.

The epidermal growth factor receptor (EGFR) was discovered by Stanley Cohen, and is a member cell-surface receptor of the ErbB family, a subfamily of four closely related receptor tyrosine kinases, including EGFR (ErbB-1), HER2/c-neu (ErbB-2), Her 3 (ErbB-3) and Her 4 (ErbB-4) [42–45]. EGFR exists on the cell surface and is activated by binding of its specific ligands, including epidermal growth factor and transforming growth factor α (TGFα). Upon activation by its growth factor ligands, EGFR undergoes a transition from an inactive monomeric form to an active homodimer [46]. In addition to forming homodimers after ligand binding, EGFR may pair with another member of the ErbB receptor family, such as ErbB2/Her2/neu, to create an activated heterodimer [47]. EGFR dimerization stimulates its intrinsic intracellular protein-tyrosine kinase activity. As a result, autophosphorylation of several tyrosine (Y) residues in the C-terminal domain of EGFR occurs [48]. This autophosphorylation elicits downstream activation and signaling by several other proteins that associate with the phosphorylated tyrosines through their own phosphotyrosine-binding SH2 domains. These downstream signaling proteins initiate several signal transduction cascades, such as MAPK, Akt and JNK pathways, leading to a serial gene expression, by which mediates alteration of cellular function, such as cell migration, adhesion, proliferation and transformation. It has been reported that mutations leading to EGFR constant activation could also produce uncontrolled cell division [49]. The somatic mutations leading to EGFR overexpression or over-activated have been associated with a number of cancer development, progression, angiogenesis and metastatic spread [50–52]. Dysregulation and/or amplification of the EGFR gene and/or mutations in the EGFR tyrosine kinase domain are known to be implicated in about 30% of all epithelial cancers [8–11]. Thus, the EGFR is becoming a dominant target for scientists attempting to understand cancer and for clinicians attempting to improve cancer treatment, including using specific tyrosine kinase inhibitors (TKI) and monoclonal antibodies specific targeting EGFR. However, many patients develop resistance to those therapeutic drugs. Therefore, investigating and understanding of the EGFR upstream modulatory mechanisms might provide some novel targets for cancer therapy. p85α is a multifunctional protein and serves as a critical mediator in various physiological processes, via either PI3K-dependent or -independent mechanisms [25, 53, 54]. One previous study reports the involvement of p85α in the p53-mediated apoptotic response to oxidative stress, which is unrelated to the activation of the PI3K signal transduction pathway, suggesting the potential role of p85α in transmitting cell damage signaling [55], while our published studies also demonstrate that p85α plays a critical role in mediating UV-induced apoptosis through the induction of TNFα gene expression and this special pro-apoptotic effect of p85α is unrelated to the PI3K-dependent signaling pathway [26]. We further demonstrate that the inducible transcription factor NFAT3 is the major downstream target of p85α for mediating UV-induced TNFα transcription [26]. Our current study found that EGFR can be regulated by p85α through C-Jun-mediated transcriptional activation of nucleolin, which could bind to and stabilize EGFR mRNA and subsequently resulted in EGFR protein expression, and further in turn promoting cell transformation following EGF treatment. This novel finding of new function of p85α and its regulated nucleolin could potential serve as new targets for cancer prevention and therapy.

Nucleolin (NCL), a ubiquitously expressed acidic phosphoprotein with key functions both in transcription and in the synthesis and maturation of ribosomes [56]. NCL was originally identified as a nuclear protein localizing primarily to the nucleoli, but is now appreciated to undergo nuclear-cytoplasmic shuttling and to also be present on the cell surface of some types of cells [57, 58]. NCL has been found to bind to the mRNA of several important genes, including p53 [59], bcl-2 [60], and bcl-xl [61], leading to regulation on mRNA turnover or translation, NCL is therefore involved in critical aspects of gene expression regulation, by which modulates cell proliferation, cell growth and many other cellular function as well [62, 63]. Inhibition of cell-surface NCL and NCL activities suppresses cell and tumor growth in breast, prostate, and glioma cell lines [64]. An aptamer-targeting NCL, AS1411, is in phase II clinical trial for relapsed/refractory acute myeloid leukemia [65], metastatic renal cell carcinoma [66] and malignant melanoma [67]. Previous study has demonstrated that NCL can bind EGFR protein to enhance EGFR activation [68]. Here we found that p85α upregulated NCL expression, and the upregulated-NCL consequently mediated EGFR expression and EGF-induced cellular malignant transformation. Regulation of mRNA stabilization is one of the major mechanisms responsible for cells controlling protein expression and is regulated by multiple proteins [15, 16]. The defect in regulation of mRNA stability might lead to complicated disorders, including cancers [18]. Rates of mRNA degradation in the cytoplasm are regulated by the sequences of the nucleic acid (cis-elements on the mRNA) and the proteins that bind to them (trans-acting factors) [69]. The most well-characterized mRNA cis-elements are AU-rich sequences [70]. There are distinct classes of AU-rich elements (AREs), ranging from arrays composed of several AUUUA elements in oncogene mRNAs to individual AUUUA elements scattered in 3′-UTR sequences of EGFR mRNAs [71, 72]. Our studies here revealed that p85α-upregulated NCL can bind to EGFR mRNA and subsequently elevating EGFR mRNA stability, further facilitating EGF-induced cellular malignant transformation, as well as cell migration (Supplementary Figure 1). During our studies, we also found that NCL is regulated by p85α at transcription level, rather than mRNA degradation level. Although Bioinformatics analysis indicated that there are a series of the putative transcription factor binding sites, including specificity protein (Sp)-1, CREB, E2F and AP-1 in the promoter regions of the NCL. The results obtained from comprehensive investigations demonstrated that AP-1 (C-Jun) was crucial for p85α-initiated nucleolin transcription, and its downstream biological effects.

In summary, our current studies revealed a novel link between p85α and EGFR mRNA stability and EGFR upregulation through p85α-initiated and C-Jun-mediated nucleolin transcriptional activation. The newly identified NCL directly binding to and interacting with EGFR mRNA and stabilizing EGFR mRNA and its expression is further responsible for promotion of EGF-induced malignant cell transformation and migration. These p85α-related new findings provide an insight into understanding of the new face of p85α in tumorigenesis, suggesting that p85α could potentially be used as a preventive/therapeutic target for cancers.

## MATERIALS AND METHODS

### Reagents, antibodies and plasmids

Actinomycin D (Act D) and EGF were purchased from Calbiochem (San Diego, CA) and Promega (San Luis Obispo, CA, USA), respectively. The dual luciferase assay kit was obtained from Promega (Madison, WI, USA). TRIzol reagent and SuperScriptTM First-Strand Synthesis system were bought from Invitrogen (Grand Island, NY, USA). PolyJetTM DNA *In vitro* Transfection Reagent was purchased from SignaGen Laboratories (Rockville, MD, USA). The specific antibody for p85α was purchased from Abcam (Cambridge, MA, USA). NCL, HUR, α-Tubulin, p300 and E2F1 antibodies were purchased from Santa Cruz Biotechnology, Inc. (Santa Cruz, CA, USA), EGFR, C-Jun, p65, CREB and p-CREB antibodies were purchased from Cell Signaling Technology (Beverly, MA, USA), AUF1 antibody was purchased from Aviva (San Diego, CA, USA). GAPDH antibody was purchased from Gene Tex (Irvine, CA, USA). The EGFR promoter-luciferase reporter, in which the transcription of the luciferase reporter gene is driven by the up-stream 5′- flanking region of the EGFR, and NCL promoter-Luciferase reporter (nucleotides −1260 to +60), had been described previously [73]. EGFR-GFP was obtained from Addgene (plasmid 32751). GFP-NCL expression vector was a generous gift from Dr. Michael B. Kastan (Duke University School of Medicine, Duke Cancer Institute, Durham, NC) [59]. GFP-HUR expression vector was a generous gift from Dr. Imed-Eddine Gallouzi (McGill University Health Center, McGill University, Montreal, Canada) [74]. The plasmid of C-Jun was used and is described in our previous study [75]. The shRNAs specific targeting p85α, HUR, and NCL, were bought from Open Biosystems (Huntsville, AL, USA).

### Cell culture and transfection

p85α+/+ and p85α−/− cells were isolated from wild-type and p85α−/− mice and described in a previous study [26]. The cells were maintained in DMEM (Invitrogen, Carlsbad, CA, USA) supplemented with 10% FBS (Nova-Tech, Grand Island, NE, USA), 1% penicillin/streptomycin, and 2 mM L-glutamine (Life Technologies, Grand Island, NY, USA) at 37°C in 5% CO_2_ incubator. Cell transfections were performed by using PolyJetTM DNA *In vitro* Transfection Reagent, according to the manufacturer's instruction. The stable transfectants of shp85α, shHUR, shNCL in p85α+/+ cells were selected in culture medium containing 5 μg/mL puromycin (Alexis, Plymouth, PA) and the resultant stable transfectants were identified for desired protein expression. And the stable transfectants of EGFR, HUR, NCL, C-Jun in p85α−/− cells were selected by 2.5 μg/ml blasticidin (Fisher Scientific, Pittsburgh, PA).

### Luciferase reporter assay

MEF cells were co-transfected with the EGFR- or NCL promoter-luciferase reporter constructs, together with the Renilla luciferase vector pRL-TK (Promega, Madison, WI). After stabilization, the cells were treated with passive lysis buffer according to the dual-luciferase assay manual (Promega), and then measured with a luminometer (Lumat LB9507, Berthold Tech., Bad Wildbad, Germany). The firefly luciferase signal was normalized to the Renilla luciferase signal for each individual analysis to eliminate the variations of transfection efficiencies as previously described [73].

### Soft agar colony formation assay

Soft agar assay was performed according to the protocol described previously [76]. Briefly, the cells were suspended in 1 ml of medium containing 0.33% agar and applied onto 3 ml of pre-solidified 0.6% agar plus 10% FBS in 6-well plates (1 × 10^4^ cells/well) with or without EGF (60 ng/ml). After about 3 weeks of incubation, colonies were observed under a phase contrast microscope, photographed and counted. The results were expressed as the mean ± S.D. of triplicate experiments.

### Reverse transcription-PCR (RT-PCR)

Total RNA was extracted with Trizol reagent. 5 μg total RNA was used for first-strand cDNA synthesis with oligdT primer by SuperScriptTM First-Strand Synthesis system (Invitrogen). Specific primer pairs were designed for amplifying murine HUR (forward: 5′-AAG AGG CAA TTA CCA GTT TCA-3′, backward: 5′-CTT CAT AGT TTG TCA TGG TCA C-3′), EGFR (forward: 5′-GAG AGG AGA ACT GCC AGA A-3′, backward: 5′-GTA GCA TTT ATG GAG AGT G-3′), NCL (forward: 5′-GGA GGT TGT CAT CCC TCA GA-3′, backward: 5′-TCC TCC TCA GCC ACA CTC TT-3′), GAPDH (forward: 5′-TGC AGT GGC AAA GTG GAG ATT-3′, backward: 5′-TTT TGG CTC CAC CCT TCA AGT-3′). The PCR products were separated onto 3% agarose gels, stained with ethidium bromide (EB), and the images scanned with a UV light as described previously [77].

### Immunoblotting assay

Whole-cells were washed with ice-cold PBS, and then extracted with cell lysis buffer (10 mM Tris-HCl, pH 7.4, 1% SDS, 1 mM Na_3_VO_4_, and proteasome inhibitor). Cytoplasmic and nuclear proteins were prepared with the Nuclear/Cytosol Fractionation Kit (BioVision, Mountain View, CA, USA) following the manufacturer's protocols. The cell extracts were subjected to Western blotting with each of the antibodies. The protein bands specifically bound to the primary antibodies were detected using an alkaline phosphatase-linked secondary antibody and ECF (enhanced chemifluorescence) western blotting analysis system (Amersham Pharmacia Biotech, Piscataway, NJ) as previously described [78]. The results shown were representative of at least three independent experiments.

### RNA-IP assay

RNA-IP assay was performed as described previously [79]. Briefly, 293T cells were cultured in 10-cm dishes. When cell confluence reached 70∼80%, cells were transiently transfected with GFP-NCL and its GFP vector control. Twenty four hours after the transfection, the cells were extracted by using polysomelysis buffer (10 mM HEPES pH 7; 100 mM KCl; 5 mM MgCl2; 25 mM EDTA; 0.5% IGEPAL; 2 mM DTT; 50 units/ml RNase OUT; 50 units/ml Superase IN; 0.2 mg/ml heparin; and complete proteinase inhibitor). The cell lysates were centrifuged at 14,000 × g for 10 min at 4°C. The anti-GFP agarose beads A/G (Vector laboratories, Burlingame, CA, USA) were added into the supernatant and rotated overnight at 4°C in NET2 buffer (50 mM Tris-HCl, pH 7.4, 150 mM sodium chloride, 1 mM magnesium chloride, 0.05% IGEPAL, 50 U/mL RNase OUT, 50 U/mL Superase IN, 1 mM dithiothreitol, and 30 mM EDTA). The beads were washed three times, and resuspended in 100 μL NET2 and 100 μL SDS-TE (20 mM Tris-HCl, pH 7.5, 2 mM EDTA, and 2% sodium dodecyl sulfate) and then incubated at 55°C for 30 min, mixing occasionally. The RNAs in the buffer of the beads were extracted by phenol-chloroform-isoamyl alcohol and RT-PCR was performed to identify the mRNA presented in the immune-complex.

### Statistical analysis

The student's *t*-test was used to determine the significance between treated and untreated group. The results are expressed as mean ± SD from at least three independent experiments. *P* < 0.05 was considered as a significant difference between compared groups.

## SUPPLEMENTARY MATERIALS FIGURE


