# Supplementary material for: p85α promotes nucleolin transcription and subsequently enhances EGFR mRNA stability and EGF-induced malignant cellular transformation
Source: Oncotarget. 2016 Feb 24;7(13):16636–49. doi: 10.18632/oncotarget.7674 (PMC4941340; doi:10.18632/oncotarget.7674)
Supplement: Supplementary file 1 [file oncotarget-07-16636-s001.pdf]

p85α promotes nucleolin transcription and subsequently enhances EGFR mRNA stability and EGF-induced malignant cellular transformation

Supplementary Materials

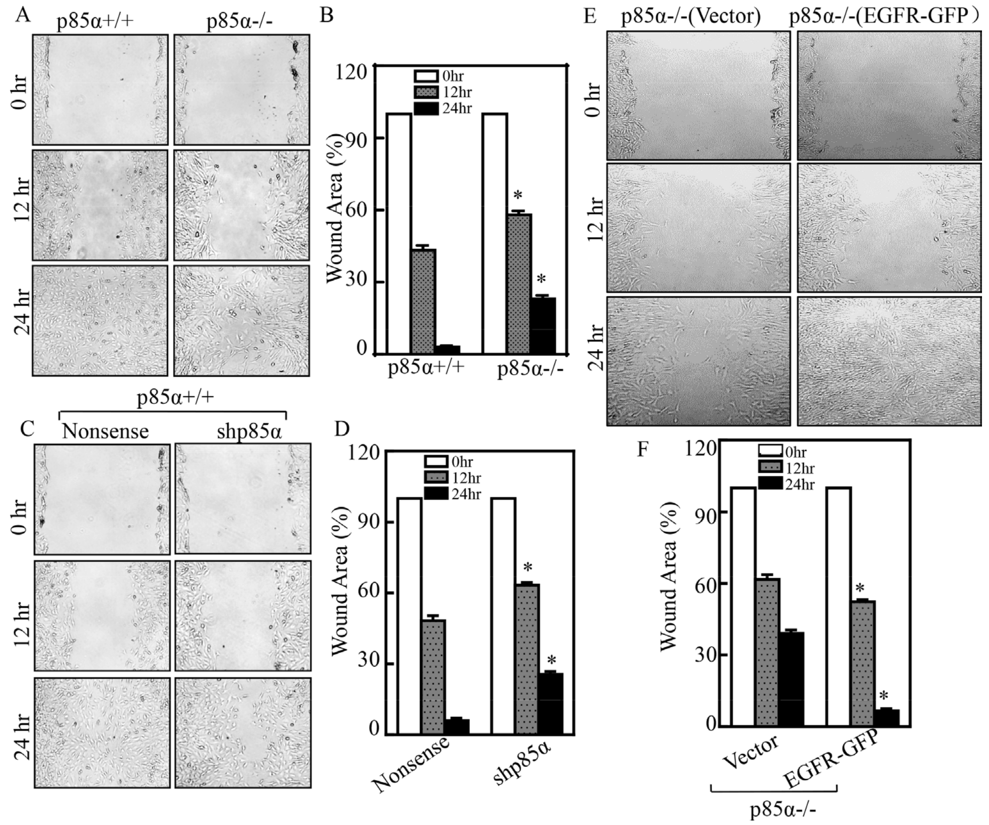

Supplementary Figure S1: p85α regulated cellular migration. Cell migration was determined by wound healing assays at the indicated times between p85α+/+ vs. p85α−/− (**A and B**), p85α+/+ (Nonsense) vs. p85α+/+(shp85α) (**C and D**) or p85α−/− (Vector) vs. p85α−/−(EGFR-GFP) (**E and F**). The wound area was quantified by using Cell Migration Analysis software, and the quantitative data was shown as indicated (error bar represent SD, *n* =3). The asterisk (*) indicates a significant difference between the indicated two cell lines ($P < 0.05$).
